# Male sex is associated with aggressive behaviour and poor prognosis in Chinese papillary thyroid carcinoma

**DOI:** 10.1038/s41598-020-60199-9

**Published:** 2020-03-05

**Authors:** Jinhua Ding, Weizhu Wu, Jianjiang Fang, Jing Zhao, Li Jiang

**Affiliations:** 1 Department of Breast and Thyroid Surgery, Ningbo Medical Center Lihuili Hospital, Ningbo, China; 2 Department of Breast and Thyroid Surgery, Taipei Medical University Ningbo Medical Center, Ningbo, China; 3 Department of Emergency, Ningbo Medical Center Lihuili Hospital, Ningbo, China; 4 Department of Emergency, Taipei Medical University Ningbo Medical Center, Ningbo, China; 5 Department of General Practice, Ningbo Medical Center Lihuili Hospital, Ningbo, China; 6 Department of General Practice, Taipei Medical University Ningbo Medical Center, Ningbo, China

**Keywords:** Head and neck cancer, Thyroid diseases

## Abstract

The differences in prognosis of papillary thyroid carcinoma (PTC) by sex have been investigated in several previous studies, but the results have not been consistent. In addition, the impact of sex on the clinical and pathological characteristics, especially on central lymph node metastasis (CLNM), still remains unknown. To the best of our knowledge, the impact of sex on PTC has not been investigated in the Chinese PTC population. Therefore, our study retrospectively analysed the data of 1339 patients who were diagnosed with PTC and had received radical surgery at Ningbo Medical Center, Lihuili Hospital. In addition to cancer-specific death, structural recurrence and risk stratification, prognosis was also estimated by using three conventional prognostic systems: AMES (age, distant metastasis, extent, size), MACIS (distant metastasis, age, completeness of resection, local invasion, size) and the 8^th^ version TNM (tumor, lymph node, metastasis) staging system. The clinical and pathological characteristics and above prognostic indexes were compared between male and female PTC patients. The results showed that there were higher rates of non-microcarcinoma PTC (nM-PTC), CLNM, lateral lymph node metastasis (LLNM), advanced disease and bilateral disease, but there was a lower rate of concurrent Hashimoto’s thyroiditis (HT) in male PTC patients than in female PTC patients. Additionally, the rate of intermediate-risk, high-risk or advanced disease was higher in male PTC patients. The above findings indicate that PTC in men is a more aggressive disease and may have a worse prognosis; thus, it should be treated with more caution.

## Introduction

In some cancers that occur in both sexes, such as thyroid, lung and liver cancer, there is an obvious difference in incidence between sexes^1–3^. Thyroid cancer is a malignancy of the endocrine system that has been rapid-growing in recent years, and it was reported that the incidence of thyroid cancer in women and men was 13.5 and 3.70 per 100,000 persons, respectively, in China in 2012^4^.

Previous studies demonstrated that PTC in men had a worse prognosis than in women^2,5^, which was easily to be explained by epidemiologic data. The ratio of the mortality rate to the incidence rate, also called as the death/incidence ratio, is a useful index to indicate the prognosis of a disease. Since the estimated incidence rate of PTC in women was 3-fold higher than that in men^6,7^, and the estimated death rate in women was only 1.3-fold higher than that in men^7^, the death/incidence ratio in men should be higher than that in women. However, Yorke E *et al*. found a similar prognosis in men and in women when the influencing factors including patient age, tumour size, lymph node status, and extrathyroidal extension were balanced^8^.

However, the above studies were from outside mainland China. Additionally, the influence of sex on clinical and pathological characteristics, especially on central lymph node (CLN) status, is currently unclear. The 2009 and revised 2015 versions of the American Thyroid Association (ATA) guidelines both strongly recommended that thyroidectomy without prophylactic central neck dissection is appropriate for small (T1 or T2), noninvasive, clinically node-negative (cN0) PTC and for most follicular cancers^9,10^. Hence, the status of the cervical lymph node was unknown for the majority of PTCs, especially in cN0-small tumours and then TNM staging could not be determined. Our data (not published) showed that there was as high as 40% of central lymph node metastasis (CLNM) in patients with PTC. For Chinese patients with PTC, the impact of sex on the clinicopathological characteristics and long-term prognosis is still unknown. Therefore, the aim of our study was to investigate whether there were any differences in clinicopathological characteristics or estimated long-term prognosis between the sexes in a large group of Chinese patients with PTC.

## Material and Methods

### Study population

A retrospective cohort study of patients undergoing radical surgery for PTC at Ningbo Medical Center Lihuili Hospital from January 2013 to November 2015 was undertaken. All patients were diagnosed with PTC, and preoperative cervical lymph node status was determined by routine neck ultrasonography. Exclusion criteria were as follows: (1) non-papillary thyroid carcinoma; (2) distant metastasis at the initial presentation; (3) less than three CLNs removed at initial surgery; (4) incidental PTC, which was defined as PTC incidentally discovered or confirmed by microscopic examination from surgical specimens removed for the evaluation of other disease entities^11^; (5) any previous surgery on the thyroid gland or cervical lymph nodes; (6) a history of or the presence of non-thyroid head & neck malignancy; and (7) incomplete data. The study was approved by the Ethics Committee of the hospital, and each patient provided written informed consent for the study. The study protocol was conducted in accordance with the recommendations outlined in the Helsinki Declaration of 1975.

### Treatment

Surgery for PTC included the removal of the thyroid gland and regional lymph nodes. Lobectomy plus isthmectomy and total thyroidectomy (TT) were two surgical inventions used for the removal of the thyroid gland. Since 2013, in our center, prophylactic central lymph node dissection (CLND) has been routinely performed for PTC patients regardless of tumor size or central lymph node status. Lateral lymph node dissection (LLND) including levels II, III, IV and V in the lateral compartment was performed with the preoperative evidence of lateral lymph node metastasis by neck ultrasonography or cytopathology. Table 1 shows the details of the surgical treatments received by the PTC patients.

**Table 1:** Surgical treatments of 1339 PTC patients.

Surgical patterns	Number of patients	%
Lobectomy + isthmectomy + CLND	665	49.7
Total thyroidectomy + CLND	454	33.9
Lobectomy + isthmectomy + CLND + LLND	18	1.3
Total thyroidectomy + CLND + LLND	202	15.1

PTC: papillary thyroid carcinoma; CLND: central lymph node dissection; LLND: lateral lymph node dissection.

After the operation, levothyroxine was used for suppressive treatment in every patient, and the dosage was based on thyroid function. Radioactive iodine (RAI) therapy was administrated to the patients whose tumour cells had spread to extrathyroid tissue or lateral cervical lymph nodes. Levothyroxine withdrawal for 3 to 4 weeks was essential to achieve a TSH level ≥30 mIU/L before RAI therapy. The dosage of radioiodine was individualized and based on clinical experience.

All patients were routinely seen every 3 months in the first 2 years and then every 6 months in the following 3–5 years. Thyroglobulin measurement was performed every 3 months in each patient whose thyroid gland had been completely removed, and neck ultrasound was performed every 6 months.

### Data collection

PTMC was defined as a papillary thyroid carcinoma with the largest dimension equal to or less than 10 mm, regardless of its invasion or lymph node metastasis. Recurrence was defined as the development of new structural or anatomical abnormalities on imaging (high-resolution neck ultrasound), which was confirmed by fine-needle aspiration cytology or histopathology when a reoperation was performed. LLND including levels II, III, IV and V dissection was then performed if there was evidence of recurrence in the lateral compartment. The last follow-up was July 2019.

Patients’ demographics and tumour characteristics were collected from the medical records at initial surgery: sex, age, body mass index (BMI), concurrent Hashimoto’s thyroiditis (HT), thyroid function, tumour size, laterality, multifocal disease, bilateral disease and lymph node status. Additionally, the patterns of recurrence were also documented.

The study on the prognosis of PTC is challenging, because PTC may recur or metastasize over more than 10 years. Therefore, in the current study, we also used the 2015 ATA risk stratification system and three conventional prognosis systems (AMES^12^, MACIS^13^ and TNM^14^) to estimate the long-term prognosis.

### Statistical analysis

Continuous variables were analysed by *t*-test, and categorical variables were analyzed by chi- square test or Fisher’s exact test. Univariate analysis was performed to detect the associations between sex and clinicopathological characteristics. Then, a multivariate analysis including all variables with p < 0.05 in the univariate analysis, was performed to test factors’ independence by logistic regression analysis. All statistical tests were two-sided, and a p value less than 0.05 was considered as statistically significant. Odds ratios (ORs) and 95% confidence intervals (CIs) were also calculated. All statistical analyses were performed using SPSS 20.0 software (SPSS, Chicago, IL, http://www.spss.com).

## Results

### Demographic and clinicopathological characteristics

**Figure 1:**
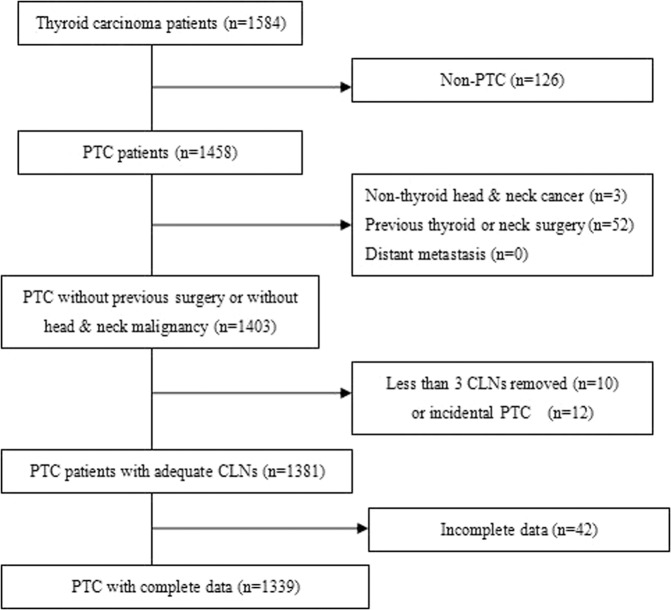
Flow chart of this study. PTC: papillary thyroid carcinoma; CLN: central lymph node.

From January 2013 to November 2015, a total of 1584 patients with PTC received radical surgery at Ningbo Medical Center Lihuili Hospital, and finally 1339 patients were included in this study. Figure 1 shows the flow chart of this study. Of the included patients, 289 (21.6%) were male and 1050 (78.4%) were female, and the age was 45.7 ± 11.2 (mean ± SD) years in this cohort.

PTMC was found in 941 (70.3%) patients, and extrathyroidal extension, multifocal disease and bilateral disease were detected in 78 (5.8%), 172 (12.8%) and 214 (16.0%) patients, respectively. A total of 653 (48.8%) patients had lymph node metastasis at their initial surgery, of whom 616 (46.0%) patients had CLNM and 210 (15.7%) patients had CLNM and LLNM. Table 2 describes the details of the included patients.

**Table 2:** Patients’ demographics features and clinicopathological characteristics.

Variable	Number of patients	%
Case number	1339	
Age (mean ± SD, years)	45.7 ± 11.2	
Groups (<55 years vs ≥55 years)	1016/323	75.9/24.1
Gender (m/f)	289/1050	21.6/78.4
BMI (≤25 vs >25 Kg/m^2^)	377/962	28.2/71.8
Hashimoto’s thyroiditis (yes/no)	233/1106	17.4/82.6
Graves’ disease (yes/no)	13/1326	1.0/99.0
Nodular goiters (yes/no)	421/918	31.4/68.6
Basic disease (yes/no)	133/1206	10.0/90.0
Malignant tumor history	13/1326	1.0/99.0
Laterality (left/right/both sites)	519/606/214	38.8/45.2/16.0
TSH level (mIU/L, mean ± SD)	2.9 ± 1.5	—
Free-T_3_ level (pmol/L, mean ± SD)	4.1 ± 1.6	—
Free-T_4_ level (pmol/L, mean ± SD)	16.2 ± 5.4	—
Tumor size (≤10 mm vs >10 mm)	941/398	70.3/29.7
Multifocality (yes/no)	177/1162	13.2/86.8
Bilaterality (yes/no)	214/1125	16.0/84.0
Extrathyroid extension (yes/no)	78/1261	5.8/94.2
Number of CLNs removed	5.8 ± 2.3	—
Number of LLNs harvest	23.8 ± 10.4	—
Lymph node metastasis (yes/no)	653/686	48.8/51.2

SD: standard deviation;

BMI: body-mass index; TSH: Thyroid Stimulating Hormone; Free-T3: free triiodothyroxine; Free-T4:free

tetraiodothyroxine; CLN: central lymph node; LLN: lateral lymph node.

### Univariate analysis and multivariate logistic regression analysis

The univariate analysis showed that there was no significant difference between the sexes regarding age, BMI, TSH level, tumor site or pathological extrathyroid extension. The rates of multifocal PTCs, bilateral PTCs, nM-PTC, CLNM, LLNM, and advanced disease were higher in male PTC than in female PTC, however, the rate of concurrent HT was lower in male PTC than in female PTC (Table 3). The multivariate logistic regression analysis found that all the above variables, except multifocal PTCs, were independent factors (Table 4).

**Table 3:** Relationship between sex and clinicopathological characteristics in 1339 PTC patients PTC: papillary thyroid carcinoma; OR: odds ratio; CI: confidence interval; BMI: body-mass index; TSH: thyroid stimulating hormone; CLNM: central lymph node metastasis; LLNM: lateral lymph node metastasis.

Variables	Male (n)	Female (n)	P value	OR	95%CI
**Age**					
<55 years	224	792	0.258	1.123	0.824–1.530
≥55years	65	258			
**BMI (Kg/m**^**2**^**)**					
≤25	205	757	0.712	0.945	0.709–1.259
>25	84	293			
**Laterality**					
Left	114	423	0.291	—	—
Right	135	516			
Left+Right	40	111			
**TSH level (mIU/L)**					
≤ 2.9	162	612	0.502	0.913	0.702–1.187
> 2.9	127	438			
**Hashimoto’s thyroiditis**					
Yes	23	210	<0.001	0.346	0.220–0.544
No	266	840			
**Tumor size (mm)**					
≤ 10	184	745	0.036	0.739	0.561–0.973
>10	105	305			
**Multifocality**					
Yes	49	128	0.039	1.471	1.027–2.105
No	240	922			
**Bilaterality**					
Yes	62	152	0.005	1.614	1.161–2.243
No	227	898			
**Extrathyroid extension**					
Yes	49	149	0.241	1.235	0.868–1.757
No	240	901			
**CLNM**					
Yes	158	458	0.001	1.559	1.200–2.025
No	131	592			
**LLNM**					
Yes	61	149	0.005	1.618	1.161–2.254
No	228	901

**Table 4:** Multivariate logistic regression analysis by sex.

Variable	B	S.E.	Wals	df	Sig.	Exp (B)	95%CI for Exp (B)	
							Lower	Upper
Tumor size	0.624	0.174	12.527	1	<0.001	1.762	1.298	2.392
Bilaterality	−0.408	0.180	5.117	1	0.024	0.665	0.467	0.947
Multifocality	−0.173	0.195	0.790	1	0.374	0.841	0.574	1.232
HT	1.104	0.232	22.590	1	<0.001	3.015	1.913	4.752
CLNM	−0.496	0.176	7.941	1	0.005	0.609	0.431	0.860
LLNM	−0.427	0.182	5.506	1	0.019	0.652	0.457	0.932

CI: confidence interval; HT: Hoshimoto’s thyroiditis; CLNM: central lymph node metastasis; LLNM: lateral lymph node metastasis. B: regression coefficient. S.E: standard error. Wals: a statistic of test, which is numerically equal to the square of B divided by S.E. df: degree of freedom. Sig: significance. Exp (B): odds ratio.

### Structural recurrences

The median follow-up time was 46 months (ranging from 26 to 72 months). There were no cancer-specific deaths, and 20 (1.5%) structural recurrences occurred in our cohort. Of the 20 recurrences, 8 (2.8%) and 12 (1.1%) recurrences were found in male and female patients, respectively, and there was a trend for significant difference, p = 0.055. Of the 20 recurrences, only one patient had a recurrence in the thyroid bed, and the other 19 patients had node recurrences. Table 5 summarizes these recurrences.

**Table 5:** Structural recurrences in19 PTC patients.

Recurrence site	Number of patients	%
Thyroid bed	1	5.0
Central compartment	1	5.0
Level 1	1	5.0
Level 2	2	10.0
Level 3	8	40.0
Level 4	6	30.0
Level 5	1	5.0

### Expectant prognostic outcome by staging system

According to the 2015 ATA risk stratification system, patients were divided into three groups: the low-risk group, the intermediate-risk group and the high-risk group. The number of patients in the corresponding groups was 231, 48 and 10 in male patients; and 938, 92 and 20 in female patients, respectively, and the difference was significant, p < 0.001.

When the AMES staging system and MACIS score system were used to evaluate the expected prognostic outcome, there was no significant difference between men and women (p = 0.339 and p = 0.282, respectively). However, the difference was significant when the 8^th^ version TNM staging system was used (p = 0.032; Table 6).

**Table 6:** Evaluation of the expectant prognostic outcome using different staging system or scoring system.

Staging	Male (n)	Female (n)	P value	OR	95%CI
**AMES staging**					
High risk	12	30	0.339	1.473	0.744–2.915
Low risk	277	1020			
**MACIS score**					
Mean	4.32 ± 0.80	4.28 ± 0.82	0.562		
<6	271	1002	0.282	0.721	0.413–1.260
≥6	18	48			
**8**^**th**^ AJCCTNM staging*					
Stage I	246	941	0.033	0.663	0.453–0.969
Stage II/III/IV	43	109			

AJCC:American Joint Committee on Cancer; TNM: tumor size, involved lymph node, distant metastasis. *Stage I is calculated as early disease, stage II, III and IV are calculated together as advanced disease.

## Discussion

PTC is a kind of malignant disease with excellent prognosis, and most local-regional recurrence and distant metastasis may occur within10 years or more of follow-ups. A median follow-up of 46 months might be considered relatively short in the setting of differentiated thyroid cancer (DTC), therefore, in our study, we not only focused on cancer-specific death and structural recurrences of these PTC patients, but also used the 2015 ATA risk stratification system and three conventional prognosis systems to estimate the long-term prognosis of PTC patients.

Our study documented the clinicopathological characteristics of a large group of Chinese patients with PTC and demonstrated that there was a higher rate of nM-PTC, CLNM, LLNM and bilateral PTC, but a lower rate of concurrent HT in men than in women. Additionally, a higher rate of advanced disease (stage II, III and IV according to the 8^th^ version ATCC TNM staging system), a higher rate of patients with intermediate or high-risk disease, and a higher rate of structural recurrencewere also found in male than in female PTC patients although not reaching statistical significance.

In our study, there were no thyroid cancer-specific deaths in the overall population, and there were 20 structural recurrences within the follow-up period. The rate of recurrence in the present study was 1.5% in the overall population, which was lower than that in a previous study^15^. The short follow-up period and the performance of prophylactic CLND may have contributed to the relatively low rate of recurrence in our study. PTC is a relatively inert cancer, and the recurrence or metastasis from PTC may occur after a long follow-up period, therefore, in our study, it was unlikely that we could capture the majority of recurrences during the median follow-up of 46 months. Additionally, prophylactic CLND in our study decreased the recurrence in the central compartment. Kruijff S *et al*. ever reported recurrence rates in the central compartment as high as 32% when prophylactic CLND was not undertaken at the initial surgery^16^. In addition, in our study, 80% (16/20) of the structural recurrences appeared in the lateral compartment, especially in the single-level lateral compartment, which indicated that selective LLND may be a reasonable option when the operation for metastatic neck lymph nodes is performed.

When the AMES system was used, the percentage of patients with high risk was 4.15% (12/289) in men and 2.86% (30/1050) in women, and the difference was not significant, p = 0.339. Similarly, when the MACIS score system was used, the mean score was 4.32 ± 0.80 in male patients and 4.28 ± 0.82 in female patients, and the difference was not significant, p = 0.562. Meanwhile, the percentage of patients with MACIS score ≥6 was 6.23% (18/289) in men and 4.57% (48/1050) in women, and the difference was not significant, p = 0.282. However, when the prognosis was estimated by the 8^th^ version TNM staging system, 14.9% male and 10.4% female patients were diagnosed with advanced disease (stage I was considered as early disease, and stage II, III and IV were considered as advanced disease), and the difference was statistically significant. That is to say, PTC in men had an estimated worse prognosis than PTC in women, because the TNM staging system was more accurate for prognosis assessment and more widely-used in clinical practice, although the AMES system or MACIS score system was supplementarily used to determine the prognosis.

The inconsistency between the 8^th^ version of the TNM staging system and the AMES system or MACIS score system could be explained by their composition. The TNM staging system contains parameters of age, tumour size, local invasion and distant metastasis, which also constitute the AMES and MACIS score systems; however, the TNM staging system also contains the status of lymph node, which is important to predict clinical outcomes in a variety of cancers (thyroid cancer also included). For example, a patient >55 years without any high-risk factors, but with cervical lymph node metastasis, was classified into the low risk group when assessed by AMES system, and had a higher possibility of scoring <6 in the MACIS score system. However, the patient was classified as having more advanced disease and a worse prognosis than patients without lymph node metastasis. In fact, this group of patients had a worse outcome than patients with similar characteristics but without lymph node metastasis.

In our study, the rates of nM-PTC (>10 mm in largest dimension) in men and women were 36.3% and 29.0%, respectively. Although the difference was statistically significant, we still believe that the difference had little impact on prognosis, which could be well explained by the weight of this factor in the prognosis system. Tumour size is a high-risk factor in prognosis systems^17–19^, but the weight assigned to this factor is not great. In the AMES system, tumours larger than 50 mm were considered as high risk, otherwise they were considered as low risk. However, the majority of PTC patients had the tumour less than 50 mm. That is, tumour size had little impact on the risk stratification of this system. In the MACIS scoring system, although tumor 10 mm and 20 mm in size seemed obviously different, under the most circumstances, tumours with different sizes were categorized into the group of total score <6, which was considered as a low-risk group with a 10-year overall survival of 98%. In the 8^th^ TNM staging system, tumour size never changed the stage in patients younger than 55 years or in patients with lymph node metastasis. Its value was particularly evident in patients older than 55 years without lymph node metastasis. For example, a patient older than 55 years with a tumour larger than 40 mm in greatest dimension but no lymph node involvement was categorized as stage II, while a tumour less than 40 mm should be categorized as stage I.

There was a higher rate of CLNM and LLNM in men with PTC than in women with PTC, which was similar to findings from previous studies^20–22^. Lymph node metastasis was not considered as a high-risk factor in the AGES and MACIS prognosis systems; however, it was the third most important parameter for prognosis in the 8^th^ version of TNM staging system, followed by distant metastasis and patient age at initial diagnosis of PTC.

Bilateral PTC was not considered as an independent prognostic factor^17–19^, and was not included in the conventional prognosis systems (AMES, MACIS and TNM). However, bilateral PTC behaved differently from common PTC, and showed aggressive behaviour, was associated with lymph node metastasis and local-regional recurrence and predicted a worse prognosis^23–25^. Our finding of a higher rate of bilateral PTC in men than in women indicated that it was a more aggressive disease; therefore, careful attention should be paid to the detection of bilateral PTC on preoperative assessment and postoperative pathological examination.

In our study, the diagnosis of HT was based on the pathology of the surgical specimen. Our study demonstrated that there was a lower rate of concurrent HT in male PTC patients than in female PTC patients, and the result was similar to that in previous studies^26–28^. Several studies have suggested a protective effect of HT in patients with PTC^29–31^, and a lower rate of HT in men maybe suggest more aggressive behaviour and possibly a worse prognosis. However, recently published literature demonstrated that there was a worse prognosis in PTC patients with thyroid autoimmunity than in those without^28,32,33^. A review summarized and updated the data on the association of these two conditions reported between 2012 and early 2018, and showed that whether HT was beneficial or harmful in thyroid cancer patients may depend on the phenotypes of infiltrative lymphocytes^34^. Infiltrative lymphocytes, such as T cells, B cells, macrophages, Th17 cells, and NK cells were related to a better prognosis in patients with PTC, while programmed death-1+ T cells, CD25+ regulatory T cells^35^, plasmacytoid DCs^36^ and double-negative T cells^37^ and macrophages with M2 phenotype^38^ seemed to be a marker for poor prognosis.

Limitations of this study should also be acknowledged. First, the retrospective nature of the study decreases the reliability of the study; thus, future prospective studies are mandatory to verify the results. Second, the population included in this study was a cohort of Chinese patients cared for in a single centre, which does introduce some selection biases; therefore, a larger number of subjects should be enrolled in a multicentre study to validate the results. Finally, there were insufficient structural recurrences and cancer-specific deaths in the short follow-up period, and whether there was a significant difference in prognosis between male and female PTC patients is still uncertain, although a nearly significant difference was demonstrated during this short follow-up. Hence, it is uncertain whether the difference in estimated long-term prognosis found in this study is the real situation, further studies with longer follow-ups are needed to validate these findings and enable a translation of our results into clinical decision-making.

Our study shows that, when compared with female PTC patients, there was more aggressive behaviour with higher rates of bilateral PTC, nM-PTC, CLNM and LLNM, lower rate of concurrent HT and an estimated worse prognosis in male PTC patients, which suggests that PTC in men may be a more aggressive disease and should be treated with more caution.
